# Supplementary material for: Yiai Fuzheng decoction inhibits triple-negative breast cancer by remodeling the immune microenvironment
Source: Front Immunol. 2025 Sep 30;16:1615631. doi: 10.3389/fimmu.2025.1615631 (PMC12518410; doi:10.3389/fimmu.2025.1615631)
Supplement: Supplementary file 2 [file DataSheet1.zip › Supplementary File 2/Treatment/D_vs_H/07_Pathway_Analysis/Pathway_Analysis/Sig_Pathways_Name/Glutathione metabolism.pdf]

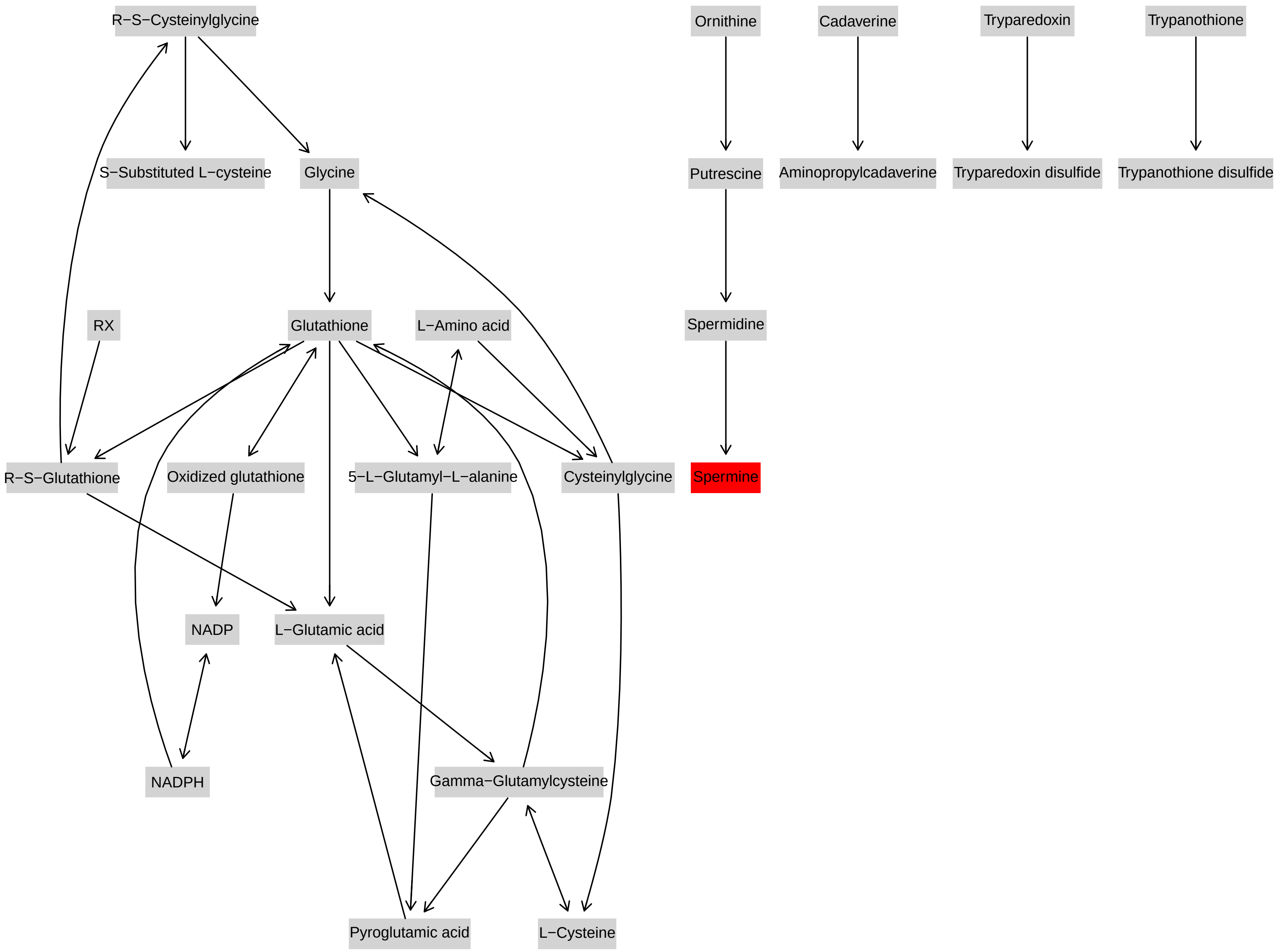
R−S−Cysteinylglycine
Ornithine
Cadaverine
Tryparedoxin
Trypanothione
S−Substituted L−cysteine
Glycine
Putrescine
Aminopropylcadaverine
Tryparedoxin disulfide
Trypanothione disulfide
RX
Glutathione
L−Amino acid
Spermidine
R−S−Glutathione
Oxidized glutathione
5−L−Glutamyl−L−alanine
Cysteinylglycine
Spermine
NADP
L−Glutamic acid
NADPH
Gamma−Glutamylcysteine
Pyroglutamic acid
L−Cysteine
